# Incidence of Electrolyte Imbalances Following Traumatic Rhabdomyolysis: A Systematic Review and Meta-Analysis

**DOI:** 10.7759/cureus.59333

**Published:** 2024-04-30

**Authors:** Saeed Safari, Seyed Hadi Aghili, Mohammad A Shahlaee, Ali Jamshidi Kerachi, Mehri Farhang Ranjbar

**Affiliations:** 1 Emergency Medicine, Shahid Beheshti University of Medical Sciences, Tehran, IRN; 2 Research Center for Trauma in Police Operations, Directorate of Health, Rescue & Treatment, Police Headquarter, Tehran, IRN; 3 Men’s Health and Reproductive Health Research Center, Shahid Beheshti University of Medical Sciences, Tehran, IRN; 4 Neurosurgery, Imam Khomeini Hospital Complex, Tehran, IRN; 5 Neurosurgery, Valiasr Hospital, Tehran, IRN; 6 Student Research Committee, Shiraz University of Medical Sciences, Shiraz, IRN; 7 Medical Surgical Nursing, School of Nursing and Midwifery, Tehran University of Medical Sciences, Tehran, IRN

**Keywords:** phosphate, calcium, sodium, potassium, meta-analysis, systematic review, electrolyte imbalance, traumatic rhabdomyolysis, rhabdomyolysis

## Abstract

Rhabdomyolysis, a medical condition caused by the destruction of striated muscle fibers, can have many etiologies, with the most common one being traumatic etiologies, that is, crushing injuries, heavy exertion, and being trapped under rubbles, and so forth. Rhabdomyolysis causes many complications, including acute kidney injury and different electrolyte imbalances, which later can cause cardiac dysrhythmia and even death as a result. This systematic review and meta-analysis investigate the incidence of imbalances of four important electrolytes among patients diagnosed with traumatic rhabdomyolysis. PubMed, Scopus, Web of Science, and Embase databases were searched for any article related to traumatic rhabdomyolysis using keywords related to the topic of our study, excluding case studies and case series. Relevant data were extracted from the included articles, and finally, a meta-analysis was performed on them to calculate the pooled incidence of each electrolyte imbalance. Collectively, 32 articles were included in our study (through the database and citation checking). The following were the pooled incidence of each electrolyte imbalance: hyperkalemia, 31% (95%CI 22%-41%); hypokalemia, 10% (95%CI 4%-17%); hypernatremia, 3% (95%CI 0%-8%); hyponatremia, 23% (95%CI 7%-44%); hypercalcemia, 0% (95%CI 0%-1%); hypocalcemia, 57% (95%CI: 22%-88%); hyperphosphatemia, 33% (95%CI 11%-59%); hypophosphatemia, 4% (95%CI 0%-16%). According to the meta-analyses, the rate of hyperkalemia, hyponatremia, hypocalcemia, and hyperphosphatemia is higher than their counterpart in patients diagnosed with traumatic rhabdomyolysis.

## Introduction and background

Rhabdomyolysis, first described by Dr. Bywaters, involves striated muscle injury, which results in the release of cell contents into the bloodstream [1,2]. The incidence of rhabdomyolysis in the United States is estimated to be around 26,000 new cases per year, although the exact frequency is unknown [3]. Rhabdomyolysis has many causes, and among the acquired causes, traumatic rhabdomyolysis is the most common [1,2,4-6].

The typical symptoms of rhabdomyolysis, regardless of its cause, include muscular weakness, myalgia, swelling, tenderness, stiffness over the affected area, tea-colored urine, oliguria, or even anuria [7]. Upon destruction of myocytes, certain substances such as myoglobin, an iron-containing molecule, and intracellular electrolytes may be released. The release of intracellular electrolytes can cause electrolyte imbalances [2] such as hyperkalemia, hypocalcemia, hyperphosphatemia, and sodium imbalances in these patients [8]. Hyperkalemia and hypocalcemia, in particular, can be life-threatening due to their impact on the cardiac conductive system and cause dysrhythmias and even cardiac arrest [5,8]. It is reported that necrosis of 100 grams of muscle can increase serum potassium levels by up to 1 mg/dL [5].

Various studies have reported the incidence of electrolyte imbalances among patients diagnosed with traumatic rhabdomyolysis; however, no meta-analysis or systematic review has been conducted on this issue. In this study, we aim to present a systematic review and meta-analysis to determine the incidence of electrolyte imbalances that have been reported to be possible in patients diagnosed with traumatic rhabdomyolysis.

## Review

Methods

Study Design and Settings

This study was designed as a systematic review and meta-analysis and conducted according to the Preferred Reporting Items for Systematic Reviews and Meta-Analyses (PRISMA) statement [9]. We investigated the incidence of electrolyte imbalances of four significant ions in the body, including sodium, potassium, calcium, and phosphorus. It is important to note that there have been no ethical considerations regarding this review. Ethical approval was not required since no individual was directly involved in this study.

Search Strategy

Databases MEDLINE (Medical Literature Analysis and Retrieval System Online) (through PubMed), Embase, Scopus, and Web of Science were searched with keywords related to traumatic rhabdomyolysis, crush injury, and crush syndrome, and also Medical Subject Headings (MeSH) terms and EmTree (also known as fixed vocabulary) in PubMed and Embase search syntax. respectively. The final designed search syntax for each database is presented in the Appendices. The initial search was conducted on July 29, 2023, and included every record until then, and the last update to our search was done on March 26, 2024. We didn’t use any filter regarding language or date to minimize publication bias.

Selection Criteria and Definitions

The primary and secondary screenings were done independently by MAS and AJK, and conflicts were solved by discussion. Rayyan AI tool for screening was used for primary screening [10]; using this tool, authors reviewed the title and abstract of every record that our search yielded. In the secondary screening phase of the study, the full text of the articles was retrieved and reviewed thoroughly. Every study on the subject of traumatic rhabdomyolysis, crush syndrome, and crush injury in which the authors reported the number of patients with any electrolyte imbalance was included. Case series, case studies, articles on rhabdomyolysis with any cause other than trauma, and articles in which the number of patients with electrolyte imbalance wasn’t reported were excluded. Following screening, duplicate articles and articles that reported findings from a shared sample were excluded. Rhabdomyolysis was defined as patients having a history of muscle injury accompanied by elevated creatine kinase (CK) level; mild rhabdomyolysis was described as having a blood creatine phosphokinase (CPK) of 300-1000 IU/L on the first day of admission, moderate rhabdomyolysis (crush injury) was defined as having blood CKP level above 1000 IU/L, crush syndrome was defined as having blood raised CPK level accompanied with systemic complication (acute kidney injury (AKI), sepsis, organ failure or respiratory failure) [7]. Normal levels for each ion are as follows: serum sodium 135-145 mEq/dL, serum potassium 3.5-5.5 mEq/dL, serum calcium 8.6-10.3 mEq/dL, and serum phosphate 2.5-4.5 mEq/dL [11,12].

Quality Assessment

MAS and AJK independently assessed the quality of the included articles using a modified JBI (Joanna Briggs Institute) critical appraisal tool for prevalence studies [13]. The JBI critical appraisal tool questionnaire has nine questions, but we didn’t include questions 3, 7, and 8. The reasons for excluding these questions from our assessment and the answers to each question are available in the Appendices. The quality assessment results are available in the Appendices.

Data Extraction

MAS and AJK used a predefined Excel sheet (Microsoft Corporation, Redmond, Washington, United States) to extract data independently. The data extracted were the authors, year of publication, cause of rhabdomyolysis, patient demographic, sample size and the number of patients diagnosed with any electrolyte imbalance, time spent under rubble (in case of earthquake), sampling method, mean blood urea nitrogen (BUN) and creatinine (Cr), and AKI presence.

Statistical Analysis

We did all eight meta-analyses using Stata Statistical Software: Release 18 (2023; StataCorp LLC, College Station, Texas, United States). Based on recently published literature, to perform a meta-analysis of single proportions (prevalence meta-analysis), it is preferred to use transformed data; for this purpose, we opted for the Freeman-Tukey Double Arcsine transformation method [14]. Using this method, we transformed raw extracted incidence data into usable effect sizes and calculated corresponding 95%CIs for each study. After preparing our meta-data, we performed meta-analyses using the random effect model (DerSimonian-Laird method) and finally reported back-transformed proportion as pooled incidence for each electrolyte imbalance (Inverse Freeman-Tukey and Clopper-Pearson exact CI).

Results

**Figure 1:**
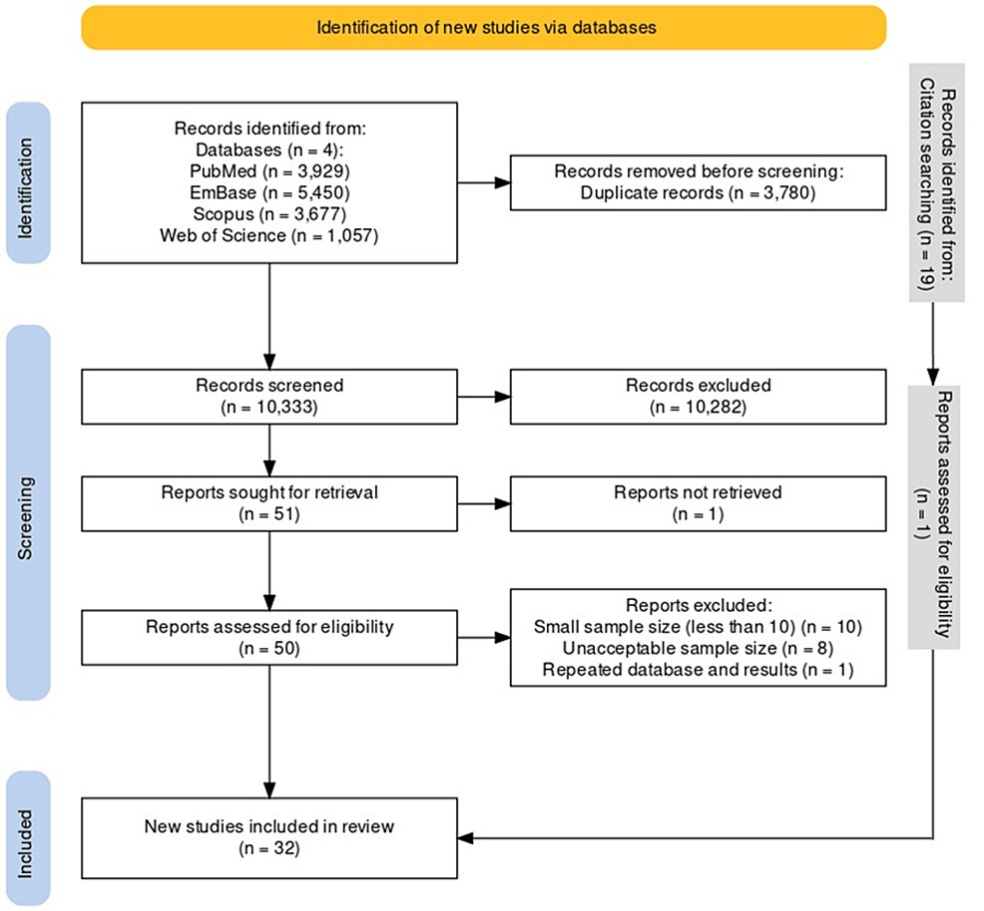
Flowchart of selection process based on Preferred Reporting Items for Systematic Review and Meta-Analyses (PRISMA) guidelines.

As shown in Figure 1, our review included 32 records in total. All included studies were retrospective descriptive studies, and their characteristics are shown in Table 1.

**Table 1:** Characteristics of included studies N/M: not mentioned, RD: retrospective descriptive study

Studies (Authors, year)	Country	Study design	Age group	Test timing
Lim et al., 2023 [15]	Singapore	RD	Adults	N/M
Safari et al., 2017 [16]	USA	RD	Adults	During hospital stay
Alafifi et al., 2023 [17]	Morocco	RD	N/M	During hospital stay
Webber et al., 2021 [18]	USA	RD	Adults	During hospital stay
Mao et al., 2021 [19]	China	RD	Adults	On admission
Thongprayoon et al., 2020 [20]	USA	RD	N/M	N/M
Luetmer et al., 2020 [21]	USA	RD	N/M	N/M
Thompson et al., 2018 [22]	USA	RD	Adults	N/M
Stewart et al., 2017 [23]	USA	RD	Adults	On admission
Safari et al., 2017 [24]	Iran	RD	Adults	On admission
Omar et al., 2016 [25]	Iran	RD	Adults	On admission
Hernández-Contreras et al., 2015 [26]	Qatar	RD	N/M	During hospital stay
Guner and Oncu, 2014 [27]	Spain	RD	N/M	N/M
Zhang et al., 2013 [28]	Turkey	RD	Adults	During hospital stay
Rosedale and Wood, 2011 [29]	China	RD	Adults	On admission
He et al., 2011 [30]	South Africa	RD	All	On admission
Bonomini et al., 2011 [31]	China	RD	N/M	On admission
Ozturk et al., 2009 [32]	Italy	RD	All	On admission
Li et al., 2009 [33]	Turkey	RD	N/M	On admission
Kang et al., 2008 [34]	China	RD	All	On admission
Aoki et al., 2007 [35]	China	RD	All	N/M
Gunal et al., 2004 [36]	Japan	RD	N/M	N/M
Sever et al., 2003 [37]	Turkey	RD	All	On admission
Demirkiran et al., 2003 [38]	Turkey	RD	All	On admission
Pocan et al., 2002 [39]	Turkey	RD	All	On hospital stay
Erek et al., 2002 [40]	Turkey	RD	All	On admission
Iskit et al., 2001 [41]	Turkey	RD	All	On admission
Naqvi et al., 1996 [42]	Turkey	RD	Pediatric	N/M
Sinert et al., 1994 [43]	Pakistan	RD	Adults	On admission
Knottenbelt, 1994 [44]	USA	RD	N/M	On admission
Malik et al., 1993 [45]	India	RD	Adults	On admission
An, 1984 [46]	China	RD	All	On admission

The sampling method used in all of these articles was the consecutive method. In the following sections, the results of our meta-analysis are presented.

Potassium Imbalance in Patients Diagnosed With Traumatic Rhabdomyolysis

After conducting our search and screening, we found 28 articles that reported the exact number of hyperkalemic patients, while 13 articles reported the number of hypokalemic patients within the studied sample (Tables 2, 3).

**Table 2:** Articles reporting the number of hyperkalemic patients among traumatic rhabdomyolysis patients ^#^: number of hypernatremic patients TUR: trapped under rubble; HE: heavy exercise

Studies (Authors, year)	Cause	Cut-off (mEq/dl)	Sample size	Number^#^	Test timing
An, 1984 [46]	Mix	6	23	20	On admission
Malik et al., 1993 [45]	Beating	Not mentioned	10	5	On admission
Knottenbelt, 1994 [44]	Beating	Not mentioned	200	8	On admission
Sinert et al., 1994 [43]	HE	5.5	35	0	On admission
Iskit et al., 2001 [41]	TUR	5.5	10	4	Not mentioned
Erek et al., 2002 [40]	TUR	Not mentioned	639	268	On admission
Pocan et al., 2002 [39]	TUR	Not mentioned	31	26	On admission
Demirkiran et al., 2003 [38]	TUR	5.5	18	7	On hospital stay
Sever et al., 2003 [37]	TUR	6	595	176	On admission
Gunal et al., 2004 [36]	TUR	Not mentioned	16	1	On admission
Aoki et al., 2007 [35]	TUR	5	345	106	Not mentioned
Kang et al., 2008 [34]	TUR	Not mentioned	49	39	Not mentioned
Li et al., 2009 [33]	TUR	Not mentioned	32	9	On admission
Ozturk et al., 2009 [32]	TUR	6	45	21	On admission
Bonomini et al., 2011 [31]	TUR	5.5	10	5	On admission
He et al., 2011 [30]	TUR	5.5	132	21	On admission
Rosedale and Wood, 2011 [29]	Beating	5	44	5	On admission
Guner and Oncu, 2014 [27]	TUR	5.5	46	43	On hospital stay
Hernández-Contreras et al., 2015 [26]	HE	Not mentioned	11	0	Not mentioned
Omar et al., 2016 [25]	Surgery	Not mentioned	17	6	On hospital stay
Safari et al., 2017 [16]	TUR	5	135	72	On admission
Stewart et al., 2017 [23]	War injury	6	778	44	On hospital stay
Thompson et al., 2018 [22]	HE	5.5	11	1	On admission
Thongprayoon et al., 2020 [20]	Heat stroke	Not mentioned	1049	68	Not mentioned
Mao et al., 2021 [19]	HE	5.5	71	0	On admission
Webber et al., 2021 [18]	HE	5.3	157	74	On hospital stay
Alafifi et al., 2023 [17]	mix	Not mentioned	35	15	On hospital stay
Lim et al., 2023 [15]	HE	Not mentioned	93	29	Not mentioned

**Table 3:** Articles reporting the number of hypokalemic patients among traumatic rhabdomyolysis patients ^#^: number of hypernatremic patients TUR: trapped under rubble; HE: heavy exercise

Studies (Authors, year)	Cause	Cut-off (mEq/dl)	Sample size	Number^#^	Test timing
Sinert et al., 1994 [43]	HE	3.5	35	0	On admission
Sever et al., 2003 [37]	TUR	3.5	595	22	On admission
Gunal et al., 2004 [36]	TUR	Not mentioned	16	9	On admission
Bonomini et al., 2011 [31]	TUR	3.5	10	0	On admission
He et al., 2011 [30]	TUR	3.5	132	24	On admission
Rosedale and Wood, 2011 [29]	Beating	Not mentioned	44	3	On admission
Guner and Oncu, 2014 [27]	TUR	3.5	46	0	On hospital stay
Hernández-Contreras et al., 2015 [26]	HE	Not mentioned	11	0	Not mentioned
Safari et al., 2017 [16]	TUR	3.5	135	5	On admission
Thompson et al., 2018 [22]	HE	3.5	11	1	On admission
Thongprayoon et al., 2020 [20]	Heat stroke	Not mentioned	1049	169	Not mentioned
Mao et al., 2021 [19]	HE	3.5	71	41	On admission
Lim et al., 2023 [15]	HE	Not mentioned	93	7	Not mentioned

Hyperkalemia Incidence

Among 28 included articles [15-20,22,23,25-27,29-41,43-46] that reported the number of hyperkalemia patients, the main cause of rhabdomyolysis was being trapped under rubble due to an earthquake. Additional details regarding the causes and features of these records can be found in Table 2. In 16 of these studies, rhabdomyolysis was diagnosed on a clinical basis [17,18,20,26,27,30,32-35,37-41,44,46], and in the rest, different serum levels of CPK were used [15,16,19,22,23,25,29,31,36,43,45]. In 16 studies, serum potassium was checked on the day of admission [16,22,29-33,36,37,39,40,43-46], it was checked during patients’ hospital stay in six of them [17,18,23,25,27,38], and in the remaining six, the authors didn’t mention anything about the timing of the test [15,20,26,34,35,41].

**Figure 2:**
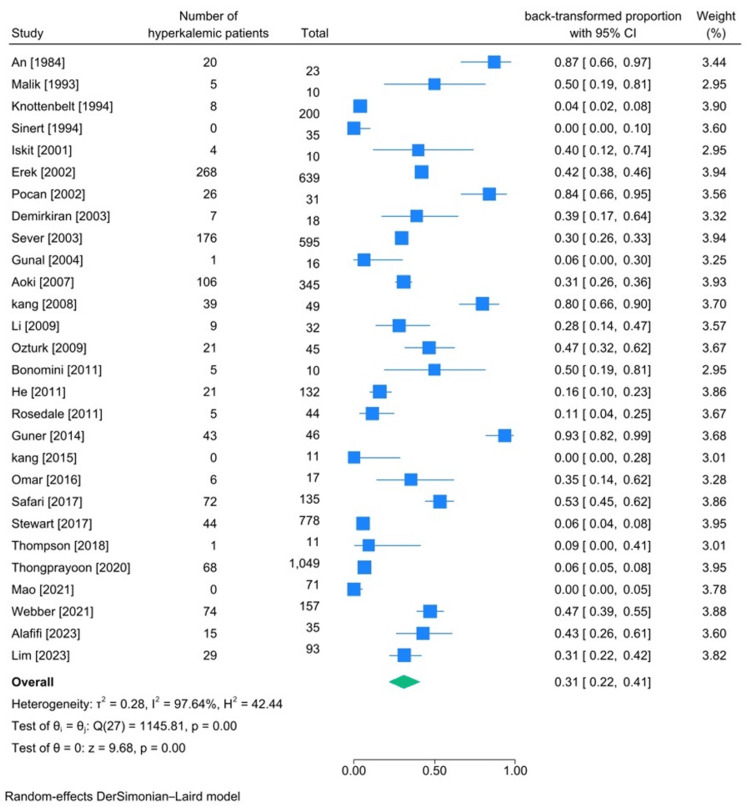
Meta-analysis result of hyperkalemia incidence among traumatic rhabdomyolysis patients References:  [15-20,22,23,25-27,29-41,43-46]

Different cut-offs for hyperkalemia were also used in these studies. The lowest observed cut-off for hyperkalemia was five mEq/dL (three articles [16,29,35]), and the highest used was six mEq/dL (four articles [23,32,37,46]). In total, hyperkalemia was assessed in 4637 patients. According to our meta-analysis, the pooled incidence of hyperkalemia among these patients was 31% (95%CI 22%-41%, heterogeneity I2:97.64%) (Figure 2).

According to a study by Migliavaca et al., a high degree of heterogeneity is expected in incidence meta-analysis (regardless of the method used) [14]. However, even with that in mind, we performed a series of subgroup meta-analyses presented in the Appendices.

Hypokalemia Incidence

**Figure 3:**
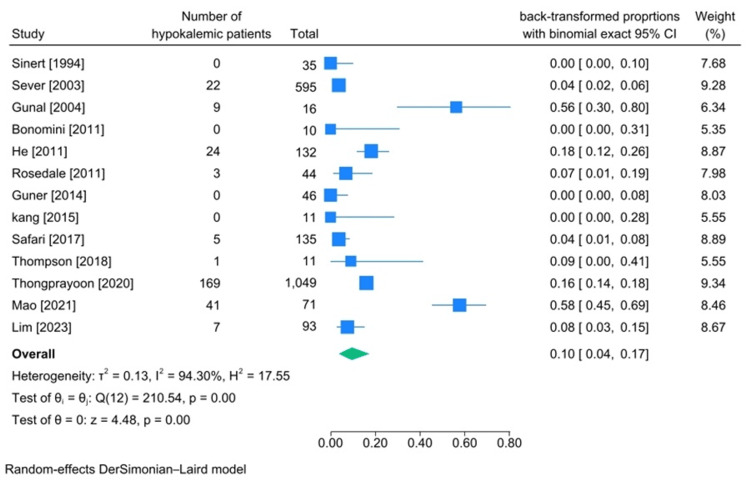
Meta-analysis on rate of hypokalemia in studies including patients diagnosed with traumatic rhabdomyolysis References: [15,16,19,20,22,24,26,27,29-31,36,37,43]

Among the 13 articles that reported the number of hypokalemia patients [15,16,19,20,22,24,26,27,29-31,36,37,43], five studied patients with rhabdomyolysis caused by exertion [15,19,22,26,43], and six reviewed rhabdomyolysis caused by being trapped under rubble after an earthquake [16,27,30,31,36,37]. In eight of these studies [16,19,22,27,30,31,37,43], hypokalemia was defined as having a serum potassium level lower than 3.5 mEq/dL, while other studies did not mention how hyperkalemia was defined. In nine of these studies, serum potassium was measured on the day of admission [16,19,22,29-31,36,37,43]. In total, 2248 patients were included in our studies. Based on our meta-analysis, the pooled incidence of hypokalemia in patients diagnosed with traumatic rhabdomyolysis was 10% (95%CI 4%-17%, heterogeneity I2:94.30%) (Figure 3).

Sodium Imbalance in Patients Diagnosed With Traumatic Rhabdomyolysis

After database search and screening, six articles were found to report the exact number of hypernatremic patients and six articles were found to convey the exact number of hyponatremic patients among their studied samples (Tables 4, 5).

**Table 4:** Articles reporting the number of hypernatremic patients among traumatic rhabdomyolysis patients ^#^: number of hypernatremic patients TUR: trapped under rubble; HE: heavy exercise; HS: heat stroke

Studies (Authors, year)	Cause	Cut-off (mEq/dl)	Sample size	Number^#^	Test timing
Sinert et al., 1994 [43]	HE	145	35	1	On admission
He et al., 2011 [30]	TUR	145	132	0	On admission
Zhang et al., 2013 [28]	TUR	145	180	11	On admission
Safari et al., 2017 [24]	TUR	145	118	8	On admission
Thongprayoon et al., 2020 [20]	HS	Not mentioned	1049	95	Not mentioned
Mao et al., 2021 [19]	HE	145	71	0	On admission

**Table 5:** Articles reporting the number of hyponatremic patients among traumatic rhabdomyolysis patients ^#^: number of hyponatremic patients TUR: trapped under rubble; HE: heavy exercise; HS: heat stroke

Studies (Authors, year)	Cause	Cut-off (mEq/dl)	Sample size	Number^#^	Test timing
Sinert et al., 1994 [43]	HE	135	35	2	On admission
He et al., 2011 [30]	TUR	135	132	45	On admission
Zhang et al., 2013 [28]	TUR	135	180	91	On admission
Safari et al., 2017 [24]	TUR	135	118	62	On admission
Thongprayoon et al., 2020 [20]	HS	Not mentioned	1049	116	Not mentioned
Mao et al., 2021 [19]	HE	135	71	1	On admission

Hypernatremia Incidence

**Figure 4:**
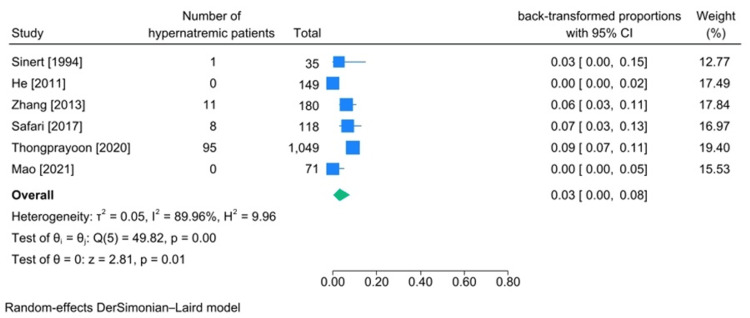
Meta-analysis on rate of hypernatremia in studies including patients diagnosed with traumatic rhabdomyolysis. References: [19,20,24,28,30,43]

Among the articles in which the number of hypernatremic patients was reported, the cause for rhabdomyolysis was heavy exertion in two of them [19,43], and in three of them, the cause of rhabdomyolysis was being trapped under rubble after an earthquake [24,28,30]. In all of these studies, serum sodium level was checked on the admission day except for one article. In total, 1585 patients were included in our analysis through these studies. The pooled incidence of hypernatremia among patients with traumatic rhabdomyolysis was calculated to be at 3% (95%CI: 0.00-0.08, heterogeneity I2:89.96%) (Figure 4).

Hyponatremia Incidence

**Figure 5:**
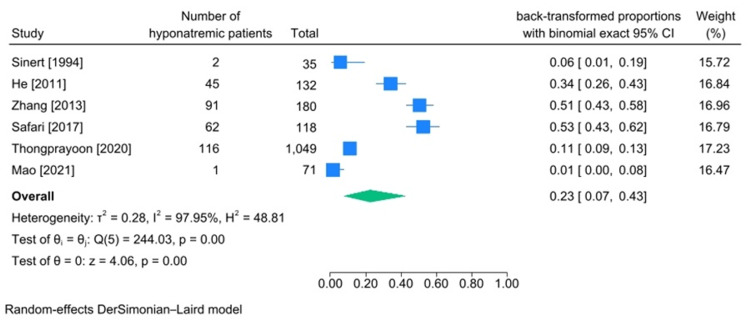
Meta-analysis on rate of hyponatremia in studies including patients diagnosed with traumatic rhabdomyolysis References: [19,20,24,28,30,43]

Six articles reported the number of rhabdomyolysis patients diagnosed with hyponatremia [19,20,24,28,30,43]. Three studied traumatic rhabdomyolysis caused by being trapped under rubble after an earthquake [24,28,30], and two articles studied patients with exertional traumatic rhabdomyolysis [19,43]. In total, 1585 patients were included in our meta-analysis through the included articles. The pooled incidence of hyponatremia in traumatic rhabdomyolysis patients was calculated to be 23% (95%CI: 0.07-0.43, heterogeneity I2:97.95%) (Figure 5).

Calcium Imbalance in Patients Diagnosed With Traumatic Rhabdomyolysis

Our meta-analyses included seven articles that reported the number of hypercalcemic patients [15,20,24,30,31,43,45] and 12 articles that reported the number of hypocalcemic patients [15,20,21,24,30,31,39-43,45] (Tables 6, 7).

**Table 6:** Articles reporting the number of hypercalcemic patients among traumatic rhabdomyolysis patients ^#^: number of hypercalcemic patients TUR: trapped under rubble; HE: heavy exercise; HS: heat stroke

Studies (Authors, year)	Cause	Cut-off (mEq/dl)	Sample size	Number^#^	Test timing
Malik et al., 1993 [45]	Beating	10.3	10	0	On admission
Sinert et al., 1994 [43]	HE	10.3	33	3	On admission
Bonomini et al., 2011 [31]	TUR	10.3	10	0	On admission
He et al., 2011 [30]	TUR	10.5	108	0	On admission
Safari et al., 2017 [24]	TUR	10.2	118	0	On admission
Thongprayoon et al., 2020 [20]	HS	Not mentioned	1049	18	Not mentioned
Lim et al., 2023 [15]	HE	Not mentioned	41	0	Not mentioned

**Table 7:** Articles reporting the number of hypocalcemic patients among traumatic rhabdomyolysis patients ^#^: number of hypocalcemic patients TUR: trapped under rubble; HE: heavy exercise; HS: heat stroke

Studies (Authors, year)	Cause	Cut-off (mEq/dl)	Sample size	Number^#^	Test timing
Malik et al., 1993 [45]	Beating	8.6	10	9	On admission
Sinert et al., 1994 [43]	HE	8.6	33	1	On admission
Naqvi et al., 1996 [42]	mix	Not mentioned	12	9	On admission
Iskit et al., 2001 [41]	TUR	Not mentioned	10	6	Not mentioned
Erek et al., 2002 [40]	TUR	Not mentioned	639	530	On admission
Pocan et al., 2002 [39]	TUR	Not mentioned	31	27	On admission
Bonomini et al., 2011 [31]	TUR	8.6	10	9	On admission
He et al., 2011 [30]	TUR	9	108	66	On admission
Safari et al., 2017 [24]	TUR	8.7	118	118	On admission
Luetmer et al., 2020 [21]	HE	Not mentioned	20	8	Not mentioned
Thongprayoon et al., 2020 [20]	HS	Not mentioned	1049	42	Not mentioned
Lim et al., 2023 [15]	HE	Not mentioned	41	0	Not mentioned

Hypercalcemia Incidence

**Figure 6:**
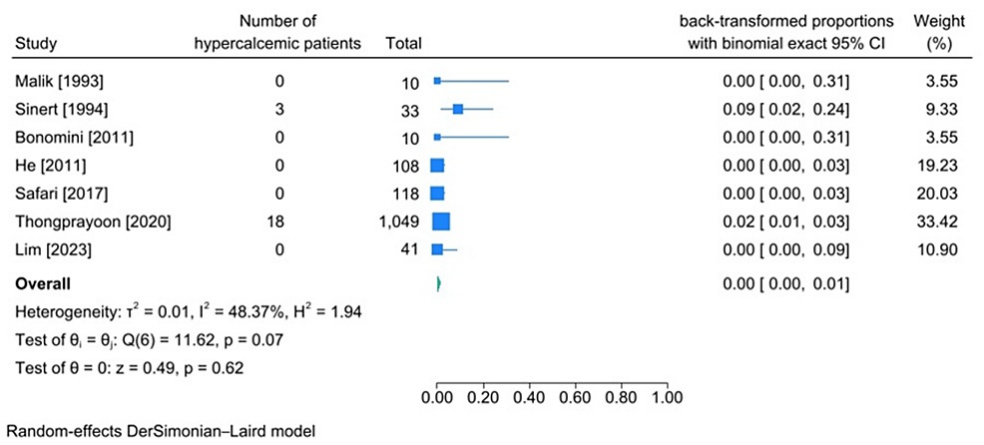
Meta-analysis on rate of hypercalcemia in studies including patients diagnosed with traumatic rhabdomyolysis. References: [15,20,24,30,31,43,45]

Among seven articles that reported the number of patients with hypercalcemia [15,20,24,30,31,43,45], the cause of rhabdomyolysis in three of them was being trapped under rubble [24,30,31], two of them were heavy exertion [15,43], one of them was being beaten [45], and one was due to heat stroke [20]. The calculated pooled incidence of hypercalcemia among patients diagnosed with rhabdomyolysis was 0% (95%CI: 0%-1%, heterogeneity I2:48.37%) (Figure 6).

Hypocalcemia Incidence

**Figure 7:**
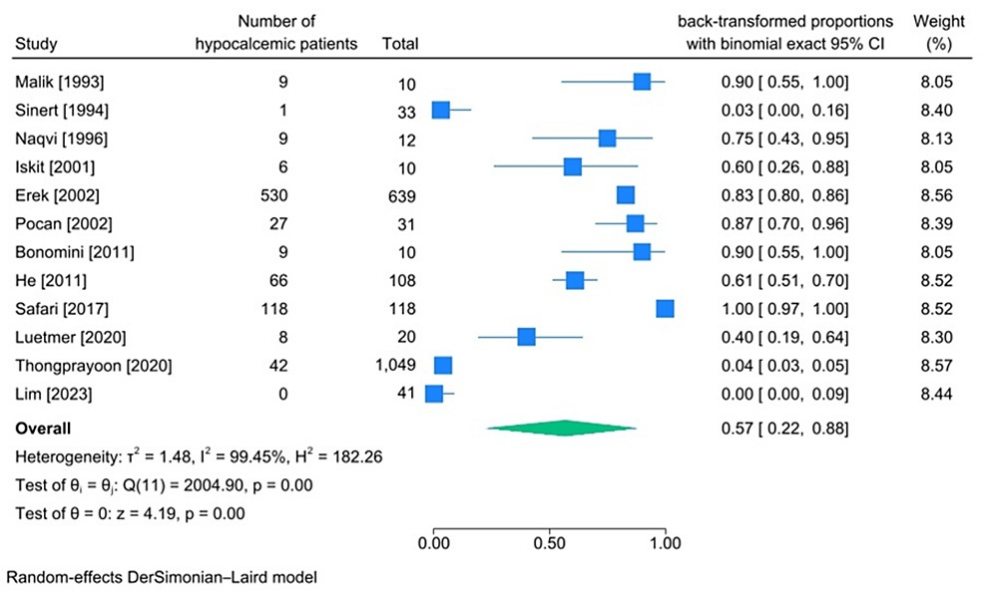
Meta-analysis on rate of hypocalcemia in studies including patients diagnosed with traumatic rhabdomyolysis. References: [15,20,21,24,30,31,39-43,45]

Among 12 articles that reported the exact number of patients diagnosed with hypocalcemia, the cause of rhabdomyolysis in six of them was being trapped under rubble [24,30,31,39-41] and in three of them, it was heavy exertion [15,21,43]. Additional details regarding the causes and features of these records can be found in Table 7. The calculated pooled incidence of hypocalcemia among patients diagnosed with rhabdomyolysis was 57% (95%CI 22%-88%, heterogeneity I2:99.45%) (Figure 7).

Phosphorus Imbalance in Patients Diagnosed With Traumatic Rhabdomyolysis

After searching databases and screening, seven articles were found to report the exact number of hyperphosphatemic patients [15,24,30,39,40,43,45], and five reported the number of hypophosphatemic patients among their studied samples [15,24,30,43,45] (Tables 8, 9).

**Table 8:** Articles reporting the number of hypophosphatemic patients among traumatic rhabdomyolysis patients ^#^: number of hypophosphatemic patients TUR: trapped under rubble; HE: heavy exercise

Studies (Authors, year)	Cause	Cut-off (mEq/dl)	Sample size	Number^#^	Test timing
Malik et al., 1993 [45]	Beating	2.5	10	4	On admission
Sinert et al., 1994 [43]	HE	2.5	18	0	On admission
He et al., 2011 [30]	TUR	Not mentioned	108	13	On admission
Safari et al., 2017 [24]	TUR	2.5	118	0	On admission
Lim et al., 2023 [15]	HE	Not mentioned	41	0	Not mentioned

**Table 9:** Articles reporting the number of hyperphosphatemic patients among traumatic rhabdomyolysis patients ^#^: number of hyperphosphatemic patients TUR: trapped under rubble; HE: heavy exercise

Studies (Authors, year)	Cause	Cut-off (mEq/dl)	Sample size	Number^#^	Test timing
Malik et al., 1993 [45]	Beating	4.5	10	0	On admission
Sinert et al., 1994 [43]	HE	4.5	18	3	On admission
Erek et al., 2002 [40]	TUR	Not mentioned	639	402	On admission
Pocan et al., 2002 [39]	TUR	Not mentioned	31	13	On admission
He et al., 2011 [30]	TUR	Not mentioned	108	19	On admission
Safari et al., 2017 [24]	TUR	3.4	118	107	On admission
Lim et al., 2023 [15]	HE	Not mentioned	41	3	Not mentioned

Hyperphosphatemia and Hypophosphatemia Incidence

**Figure 8:**
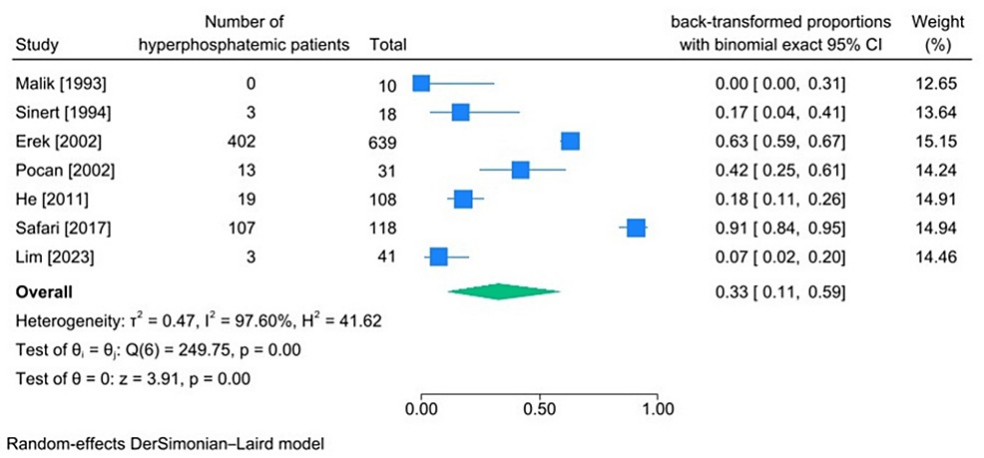
Meta-analysis on rate of hyperphosphatemia in studies including patients diagnosed with traumatic rhabdomyolysis. References: [15,24,30,39,40,43,45]

**Figure 9:**
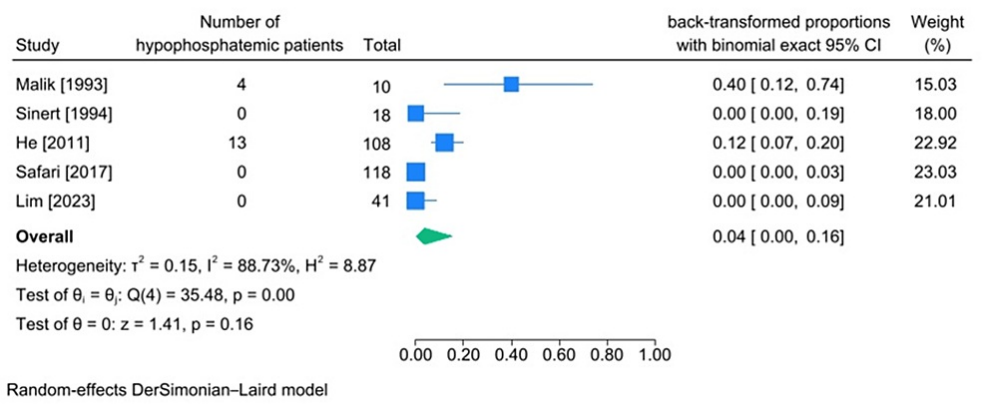
Meta-analysis on rate of hypophosphatemia in studies including patients diagnosed with traumatic rhabdomyolysis References: [15,24,30,43,45]

The characteristics of articles containing the number of patients with phosphate imbalance are presented in Tables 8, 9. The pooled incidence of hyperphosphatemia was calculated to be 33% (95%CI 11%-59%, heterogeneity I2:97.60%), and hypophosphatemia’s incidence was 4% (95%CI 0%-16%, heterogeneity I2:88.73%) (Figures 8, 9).

Discussion

This review study aimed to investigate the incidence of electrolyte imbalances in traumatic rhabdomyolysis patients. For this purpose, we conducted a systematic review and meta-analysis of previously published original articles, and to the best of our knowledge, this is the first systematic review and meta-analysis that tried to find the incidence of such complications. For the readers' convenience, this section is also divided into four categories, each representing one electrolyte imbalance.

Potassium Imbalances (Hyperkalemia and Hypokalemia)

The incidence of hyperkalemia in patients with traumatic rhabdomyolysis was found to be 31% (95%CI: 22%-41%, heterogeneity I2:97.64%). The incidence of hypokalemia among these patients was 10% (95%CI 4%-17%, heterogeneity I2 94.3%). The high degree of heterogeneity among studies included in prevalence meta-analysis has been investigated in many articles; Migliavaca et al. reviewed 134 prevalence meta-analysis articles and found that a high degree of heterogeneity is usual in such studies [14]. High I2 does not necessarily translate into true high heterogeneity among the included articles; the median of I2 of these 134 articles was 96.6% [14]. Authors have also suggested that the best way to interpret prevalence data is to discuss and explain the results with regard to what was being expected before conducting the analysis and also account for all the articles that had the highest deviation from the calculated pooled prevalence, so we tried to discuss and explain the results with this rationale.

The higher incidence of hyperkalemia among these patients compared to hypokalemia is aligned with and can be justified according to the pathogenesis of rhabdomyolysis, the process in which contents of myocytes such as potassium and phosphorus get released into the bloodstream [2,6]. Among the included articles, four articles reported a lower number of hyperkalemic patients comparatively [19,22,23,43]; three of these articles were about traumatic rhabdomyolysis due to heavy exertion [19,22,43]. Many articles state that profuse sweating during heavy exercise without replacing the lost fluid may lead to hypokalemia since electrolytes are being excreted with sweating. It is only reasonable to expect a lower incidence of hyperkalemia in this situation. One study found that the incidence of hypokalemia in a 116-patient cohort increased after exertion to 21% [47]. One of the main reasons for this occurrence is that following exertion, due to intravascular loss, the Renin-Aldosterine-angiotensin axis gets activated, causing kidneys to increase sodium reabsorption in exchange for increased potassium excretion. This mechanism eventually leads to a decrease in serum potassium, which can be an excuse for the low number of hyperkalemic patients in these three articles [5].

Stewart et al.'s study that investigated the incidence of hyperkalemia among victims of the Afghanistan and Iraq wars is another one in which the number of hyperkalemic patients reported was lower compared to pooled incidence [23]. In this study, several causes may have led to a low reported number of hyperkalemia patients. First, they defined hyperkalemia as having a serum potassium level of more than 6 mEq/dL instead of a widely used 5.5 mEq/dL cut-off. Secondly, they only investigated the victims who could survive long enough to make it to the hospital, so there may have been patients who died and were hyperkalemic. Based on the study design, when victims were being taken to the hospital in Germany (from Iraq or Afghanistan), they may have received initial therapy if they had had any signs of electrolyte imbalance. Since they reported the serum potassium level obtained within three days of the victim's hospital admission, electrolyte imbalances may have been corrected due to fluid therapy before blood tests.

Among the studies, one by An [46] and another by Guner and Oncu [27] reported the highest number of hyperkalemic patients. The authors of these two studies investigated serum potassium levels of traumatic rhabdomyolysis patients diagnosed with crush syndrome and AKI, and since AKI can itself lead to hyperkalemia, the high number of hyperkalemic patients can contribute to this. Furthermore, Guner and Oncu reported the highest recorded serum potassium levels in these patients during their hospital stay [27].

The incidence of hypokalemia in traumatic rhabdomyolysis after conducting a meta-analysis was found to be 8% (95%CI 1%-18%, heterogeneity I2:98.12%). According to the hypokalemia incidence meta-analysis forest plot diagram, Mao et al.'s study reported the highest number of hypokalemic patients [19]. They studied patients who developed traumatic rhabdomyolysis after severe exertion. As mentioned earlier, we can witness the loss of electrolytes such as potassium and other ions due to isotonic water loss. Furthermore, after conducting a leave-one-out meta-analysis, if this study gets omitted, the pooled incidence will be 3%, indicating that it had the most effect on our analysis.

Sodium Imbalances (Hypernatremia and Hyponatremia)

incidence of hypernatremia in traumatic rhabdomyolysis patients was 3% (95%CI 0%-8%, heterogeneity I2:89.96). The incidence of hyponatremia in these patients was 23% (95%CI 7%-43%, heterogeneity I2:97.95). As for the low reported number of hypernatremic patients and, accordingly, rare encounters of physicians with hypernatremia in traumatic rhabdomyolysis patients, there haven’t been many articles explaining how rhabdomyolysis can cause hypernatremia; however, according to forest plot of hypernatremia incidence, Safari et al. [24] and Zhang et al. [28] reported the highest number of hypernatremia among studied patients. In both articles, the authors studied patients who developed rhabdomyolysis after being trapped under rubble due to an earthquake. In this situation, patients may be under rubble for a long time before they get rescued and may lose water (insensible water loss). They also may be bleeding, which may eventually lead to vasopressin secretion (some studies have stated that in these situations, there is stress-induced secretion of vasopressin), which may cause hypernatremia [24]. In contrast, there are multiple explanations and reasons for hyponatremia in traumatic rhabdomyolysis. In rhabdomyolysis following myocyte damage, cell membrane functionality gets disrupted, resulting in an influx of sodium ions into cells, which draws water in (third-spacing) [5]. On the other hand, the stress of being trapped under rubble promotes vasopressin secretion, which enhances water resorption in the kidneys. Above all, myoglobin toxicity in kidneys may cause acute renal failure, leading to water overload due to the kidney’s inability to excrete water. All of the mentioned reasons may eventually lead to hyponatremia. With all that in mind, two of the included studies reported a lower incidence of hyponatremia among their studied population (Sinert et al. [43] and Mao et al. [19]). The cause of rhabdomyolysis in both of these studies was heavy exertion. We can assume muscle injury isn’t as severe in these populations as it is in patients with multiple limb injuries due to being trapped under rubble, so we can conclude less severe trauma may cause less severe complications as well since injured muscle mass directly influences the amount of third-spacing [48].

Calcium Imbalances (Hypercalcemia and Hypocalcemia)

The incidence of hypercalcemia among the patients was found to be 0% (95%CI 0%-1%, heterogeneity I2:48.37%). However, the incidence of hypocalcemia was quite high at 57% (95%CI 22%-88%, heterogeneity I2:99.45%). Medical literature provides several explanations for hypocalcemia in rhabdomyolysis patients, especially during the early stages of the disease. Firstly, the damage inflicted on muscle cell membranes (sarcolemma) causes a loss of cell membrane selective permeability, and calcium ions influx into cells, leading to a decrease in serum calcium level. Secondly, phosphate ions leak into the extracellular space, which binds to free calcium ions, augmenting renal calcium excretion. Thirdly, free calcium can bind to phosphates in damaged muscle tissue and deposit in that area. Fourthly, due to probable AKI, the production of active vitamin D3 may be interrupted, leading to decreased renal calcium reabsorption. Finally, some studies suggest that bone response to parathyroid hormone is altered in these patients, which may further worsen hypocalcemia. Of the articles included, the studies by Sinert et al. [43] and Lim et al. [15] reported the lowest number of hypocalcemic patients. This can be attributed to the fact that the degree of hypocalcemia in patients with rhabdomyolysis is closely linked to the amount of damaged muscle tissue. The studies mentioned showed that the cause of traumatic rhabdomyolysis in these patients was excessive exertion, which means that it is logical to have a lower number of hypocalcemic patients compared to other causes of traumatic rhabdomyolysis since the total injured muscle is usually less [2,5,7].

Phosphate Imbalances (Hyperphosphatemia and Hypophosphatemia)

Among investigated electrolyte imbalances, the number of patients diagnosed with phosphate imbalance was the lowest. With that being said, the incidence of hyperphosphatemia was 33% (95%CI 11%-59%, heterogeneity I2:97.6%) and the incidence of hypophosphatemia among these patients was 4% (95%CI 0%-16%, heterogeneity I2:88.73%). The pathophysiological mechanism behind this disease can justify the higher incidence of hyperphosphatemia among patients diagnosed with traumatic rhabdomyolysis since phosphate is an intracellular ion (intracellular anion). Thus, upon muscular damage, logically, it gets released into the stream, raising serum phosphate levels [5].

To sum up, hyperkalemia, hyponatremia, hypocalcemia, and hyperphosphatemia are more common among traumatic rhabdomyolysis patients. However, diagnosing traumatic rhabdomyolysis is not straightforward, as there is no agreed-upon diagnostic method. Different CPK cut-offs were used in different studies (500, 1000, and 5000), and some physicians relied on clinical symptoms to diagnose rhabdomyolysis. Additionally, various definitions of electrolyte imbalances were used, and not all patients could be investigated due to the emergency nature of the disease. Despite these limitations, we made an effort to be as inclusive and comprehensive as possible by reviewing all relevant articles

Limitations

It is important to note that our study has a few limitations. Firstly, during our primary review, we found that many studies reported the mean serum value of electrolytes for patients instead of the number of patients with imbalanced electrolyte levels. This limited the number of articles we could include in our analysis. Secondly, we were unable to include any prospective studies in our analysis because there were none on traumatic rhabdomyolysis, likely due to the urgent nature of the condition. Another limitation is that some of the included studies had small sample sizes, which can result in high heterogeneity and CIs. This issue is to be expected, as the authors of these studies did their best to include as many patients as possible within the limited resources of an emergency setting. Additionally, some patients with mild injuries may not have been evaluated or tested for electrolyte imbalances and, hence, were not included in the study. This could explain the smaller sample sizes in some of the articles.

## Conclusions

Our meta-analyses and reviews have shown that there is a logical correlation between electrolyte imbalances and traumatic rhabdomyolysis in patients. It has been observed that certain electrolyte imbalances are more prevalent in patients diagnosed with traumatic rhabdomyolysis in the early stages of the disease. This has been attributed to the fact that patients were tested during the early phase of their disease in most of the studies. The most common electrolyte imbalances in these patients include hypocalcemia (57%), hyperkalemia (31%), hyperphosphatemia (33%), and hyponatremia (23%). Conversely, electrolyte imbalances such as hypokalemia (10%), hypernatremia (3%), hypophosphatemia (4%), and hypercalcemia (in the early stages) (0%) are less prevalent and almost rare to encounter.

It is important to note that traumatic rhabdomyolysis caused by being trapped under the rubble is much more severe than traumatic rhabdomyolysis caused by exertion. Therefore, electrolyte imbalances were observed to be less common among patients who developed traumatic rhabdomyolysis after a session of heavy exertion. This is because the occurrence of complications of diseases is directly related to the severity of diseases.

## Figures and Tables

**Figure 10:**
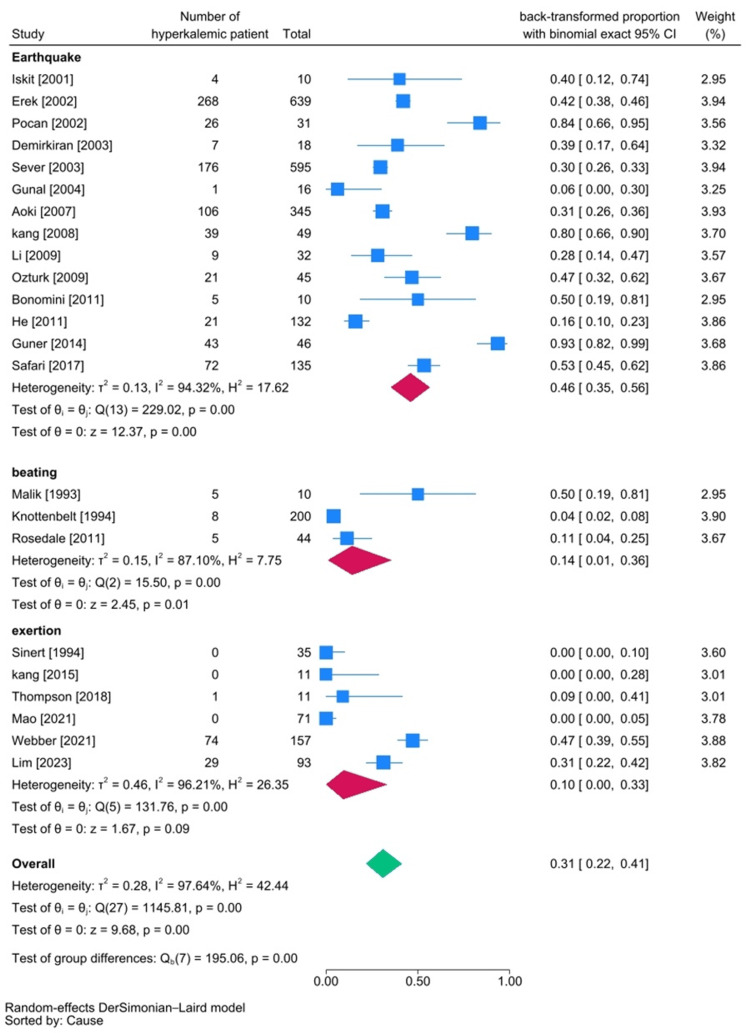
Subgroup meta-analysis based on cause of traumatic rhabdomyolysis, hyperkalemia. References: [15,16,18,19,22,23,27,29-32,35,37,38,41,43,46]

**Figure 11:**
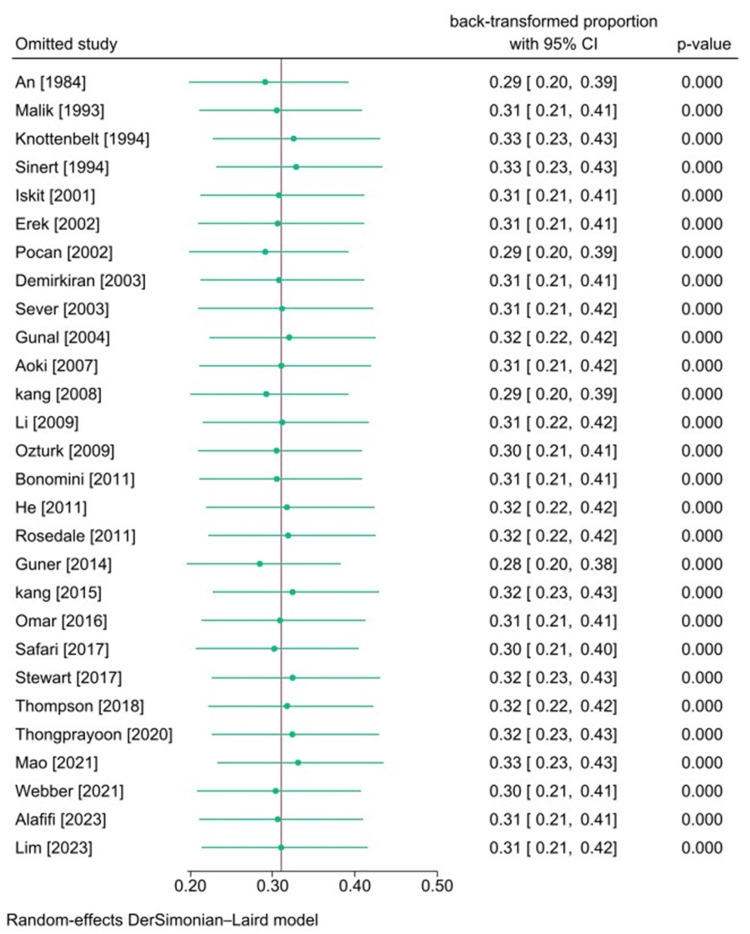
Sensitivity meta-analysis for hyperkalemia incidence among patients diagnosed with rhabdomyolysis References: [15,16,18,19,22,23,27,29-32,35,37,38,41,43,46]

## Appendices

Search queries for each database

PubMed Search Query

("rhabdomyolysis"[mh] or "compartment syndromes"[mh] or "crush injuries"[mh] or "crush syndrome"[mh] or "disasters"[mh] or "disaster victims"[mh] or "disaster medicine"[mh] or "natural disasters"[mh] or "rhabdomyolysis"[tiab] or "rhabdomyolyses"[tiab] or "rabdomiólisis"[tiab] or "traumatic rhabdomyolysis"[tiab] or "compartment syndrome"[tiab] or "compartment syndromes"[tiab] or "crush injury"[tiab] or "crush injuries"[tiab] or "crush wound"[tiab] or "crushing injury"[tiab] or "crushing trauma"[tiab] or "injuries, crush"[tiab] or "injury, crush"[tiab] or "crush syndrome"[tiab] or "crush syndromes"[tiab] or "syndrome, crush"[tiab] or "syndromes, crush"[tiab] or "exertional rhabdomyolysis"[tiab] or "exercise-induced rhabdomyolysis"[tiab] or "military casualty"[tiab] or "military casualties"[tiab] or "combat casualty"[tiab] or "combat casualties"[tiab] or "combat injury"[tiab] or "combat injuries"[tiab] or "war victim" or "war injury"[tiab] or "crush victim"[tiab] or "mass disaster"[tiab] or "disaster"[tiab] or "casualties"[tiab] or "natural disaster"[tiab] or "natural disasters"[tiab] or "battle injury"[tiab] or "battle injuries"[tiab]) and ("potassium"[mh] or "potassium/blood"[mh] or "hyperkalemia"[mh] or "sodium"[mh] or "sodium/blood"[mh] or "hypernatremia"[mh] or "hyponatremia"[mh] or "calcium"[mh] or "calcium/blood"[mh] or "hypercalcemia"[mh] or "water-electrolyte imbalance"[mh] or "water-electrolyte balance"[mh] or "electrolytes"[mh] or "electrolytes/blood"[mh] or "potassium"[tiab] or "hyperkalemia"[tiab] or "hyperkalemias"[tiab] or "hyperpotassemia"[tiab] or "sodium"[tiab] or "hypernatremia"[tiab] or "hypernatremias"[tiab] or "hypernatremia"[tiab] or "hyponatremia"[tiab] or "calcium"[tiab] or "hypercalcemia"[tiab] or "hypercalcemias"[tiab] or "water-electrolyte imbalance"[tiab] or "water electrolyte imbalance"[tiab] or "water-electrolyte imbalances"[tiab] or "water-electrolyte balance"[tiab] or "water electrolyte balance"[tiab] or "fluid balance"[tiab] or "balance, fluid"[tiab] or "electrolyte balance"[tiab] or "balance, electrolyte"[tiab] or "electrolytes"[tiab] or "serum electrolytes"[tiab] or "acute kidney injury"[mh] or "acute kidney injury"[tiab] or "acute kidney failure"[tiab]) and ("rhabdomyolysis/complications"[mesh] or "rhabdomyolysis/complications"[tiab] or "complications" [subheading] or "complications" [tiab] or "evaluation"[tiab] or "medical records"[mesh] or "medical record"[tiab] or "medical records"[tiab] or "risk assessment"[mesh] or "risk factors"[mesh] or "risk assessment"[tiab] or "risk factors"[tiab])

Scopus Search Query

(title-abs-key("rhabdomyolysis") or title-abs-key("rhabdomyolyses") or title-abs-key("rml") or title-abs-key("traumatic rhabdomyolysis") or title-abs-key("compartment syndrome") or title-abs-key("compartment syndromes") or title-abs-key("crush injury") or title-abs-key("crush injuries") or title-abs-key("crush wound") or title-abs-key("crushing injury") or title-abs-key("crushing trauma") or title-abs-key("injuries, crush") or title-abs-key("injury, crush") or title-abs-key("crush fractures") or title-abs-key("crush fracture") or title-abs-key("crushed bones") or title-abs-key("crushing fracture") or title-abs-key("fracture, crush") or title-abs-key("fractures, crush ") or title-abs-key("crush syndrome") or title-abs-key("crush syndromes") or title-abs-key("syndrome, crush") or title-abs-key("syndromes, crush") or title-abs-key("exertional rhabdomyolysis") or title-abs-key("exercise-induced rhabdomyolysis") or title-abs-key("military casualty") or title-abs-key("military casualties") or title-abs-key("combat casualty") or title-abs-key("combat casualties") or title-abs-key("combat injury") or title-abs-key("combat injuries") or title-abs-key("combat victim") or title-abs-key("war victim") or title-abs-key("war injury") or title-abs-key("crush victim") or title-abs-key("mass disaster") or title-abs-key("disaster") or title-abs-key("casualties") or title-abs-key("natural disaster") or title-abs-key("natural disasters") or title-abs-key("battle injury") or title-abs-key("battle injuries")) and (title-abs-key("potassium") or title-abs-key("hyperkalemia") or title-abs-key("hyperkalemias") or title-abs-key("hyperpotassemia") or title-abs-key("hyperpotassemias") or title-abs-key("sodium") or title-abs-key("hypernatremia") or title-abs-key("hypernatremias") or title-abs-key("hypernatremia") or title-abs-key("hyponatremia") or title-abs-key("calcium") or title-abs-key("hypercalcemia") or title-abs-key("hypercalcemias") or title-abs-key("water-electrolyte imbalance") or title-abs-key("imbalance, water-electrolyte") or title-abs-key("imbalances, water-electrolyte") or title-abs-key("water electrolyte imbalance") or title-abs-key("water-electrolyte imbalances") or title-abs-key("water-electrolyte balance") or title-abs-key("balance, water-electrolyte") or title-abs-key("water electrolyte balance") or title-abs-key("fluid balance") or title-abs-key("balance, fluid") or title-abs-key("electrolyte balance") or title-abs-key("balance, electrolyte") or title-abs-key("electrolytes") or title-abs-key("serum electrolytes") or title-abs-key("acute kidney injury”) or title-abs-key("acute kidney failye”)) and (title-abs-key("rhabdomyolysis/complications”) or title-abs-key("complications") or title-abs-key("evaluation") or title-abs-key( "medical record”) or title-abs-key(“medical records") or title-abs-key("risk assessment”) or title-abs-key("risk factors"))

Embase Search Query

(‘rhabdomyolysis’/exp or ‘crush trauma’/exp or ‘crush fracture’/exp or ‘compartment syndrome’/exp or ‘battle injury’/exp or ‘disaster’/exp or ‘accident’/exp or 'rhabdomyolysis':ab,ti or 'rhabdomyolyses':ab,ti or 'rabdomiólisis':ab,ti or 'rml':ab,ti or 'traumatic rhabdomyolysis':ab,ti or 'compartment syndrome':ab,ti or 'compartment syndromes':ab,ti or 'crush injury':ab,ti or 'crush injuries':ab,ti or 'crush wound':ab,ti or 'crushing injury':ab,ti or 'crushing trauma':ab,ti or 'injuries, crush':ab,ti or 'injury, crush':ab,ti or 'crush fractures':ab,ti or 'crush fracture':ab,ti or 'crushed bones':ab,ti or 'crushing fracture':ab,ti or 'fracture, crush':ab,ti or 'fractures, crush':ab,ti or 'crush syndrome':ab,ti or 'crush syndromes':ab,ti or 'syndrome, crush':ab,ti or 'syndromes, crush':ab,ti or 'exertional rhabdomyolysis':ab,ti or 'exercise-induced rhabdomyolysis':ab,ti or 'military casualty':ab,ti or 'military casualties':ab,ti or 'combat casualty':ab,ti or 'combat casualties':ab,ti or 'combat injury':ab,ti or 'combat injuries':ab,ti or 'combat victim':ab,ti or 'war victim':ab,ti or 'war injury':ab,ti or 'crush victim':ab,ti or 'mass disaster':ab,ti or 'disaster':ab,ti or 'casualties':ab,ti or 'natural disaster':ab,ti or 'natural disasters':ab,ti or 'battle injury':ab,ti or 'battle injuries':ab,ti) and ('potassium'/exp or 'hyperkalemia'/exp or 'electrolyte disturbance'/exp or 'hypernatremia'/exp or 'sodium'/exp or 'hyponatremia'/exp or 'calcium'/exp or 'hypercalcemia'/exp or 'hypocalcemia'/exp or 'electrolyte balance'/exp or 'potassium balance'/exp or 'sodium balance'/exp or 'electrolyte'/exp or ‘potassium’:ab,ti or ‘hyperkalemia’:ab,ti or ‘hyperkalemias’:ab,ti or ‘hyperpotassemia’:ab,ti or ‘hyperpotassemias’:ab,ti or ‘sodium’:ab,ti or ‘hypernatremia’:ab,ti or ‘hypernatremias’:ab,ti or ‘hypernatremia’:ab,ti or ‘hyponatremia’:ab,ti or ‘calcium’:ab,ti or ‘hypercalcemia’:ab,ti or ‘hypercalcemias’:ab,ti or ‘water-electrolyte imbalance’:ab,ti or ‘imbalance, water-electrolyte’:ab,ti or ‘imbalances, water-electrolyte’:ab,ti or ‘water electrolyte imbalance’:ab,ti or ‘water-electrolyte imbalances’:ab,ti or ‘water-electrolyte balance’:ab,ti or ‘balance, water-electrolyte’:ab,ti or ‘water electrolyte balance’:ab,ti or ‘fluid balance’:ab,ti or ‘balance, fluid’:ab,ti or ‘electrolyte balance’:ab,ti or ‘balance, electrolyte’:ab,ti or ‘electrolytes’:ab,ti or ‘serum electrolytes’:ab,ti or ‘acute kidney injury’/exp or ‘acute kidney injury’:ab,ti or ‘acute kidney failure’:ab,ti) and ('complication'/exp or 'risk factor'/exp or 'risk assessment'/exp or 'medical record'/exp or 'evaluation study'/exp or 'complication':ab,ti or 'risk factor':ab,ti or 'risk assessment':ab,ti or 'medical record':ab,ti or 'evaluation study':ab,ti)

Web of Science Search Query

(all=(“rhabdomyolys*”) or all=(“rabdomiólisis”) or all=(“rml”) or all=(“traumatic rhabdomyolysis”) or all=(“compartment syndrome*”) or all=(“crush* injur*”) or all=(“crush wound”) or all=(“crush* trauma”) or all=(“crush* fracture”) or all=(“crushed bones”) or all=(“crush syndrome*”) or all=(“exertional rhabdomyolysis”) or all=(“exercise-induced rhabdomyolysis”) or all=(“military casualt*”) or all=(“combat casualt*”) or all=(“combat injur*”) or all=(“combat victim”) or all=(“war victim”) or all=(“war injury”) or all=(“crush victim”) or all=(“mass disaster*”) or all=(“disaster”) or all=(“casualt*”) or all=(“natural disaster*”) or all=(“battle injur*”)) and (all=(“potassium”) or all=(“hyperkalemia*”) or all=(“hyperpotassemia*”) or all=(“sodium”) or all=(“hypernatremia*”) or all=(“hyponatremia*”) or all=(“calcium”) or all=(“hypercalcemia*”) or all=(“water*electrolyte imbalance*”) or all=(“water*electrolyte balance”) or all=(“fluid balance”) or all=(“electrolyte balance”) or all=(“electrolyt*”) or all=(“serum electrolyte*”) or all=(“acute kidney failure”) or all=(“acute kidney injury”)) and (all= ("rhabdomyolysis/complications”) or all= ("complications") or all= ("evaluation") or all= ("medical record”) or all= (“medical records") or all= ("risk assessment”) or all= ("risk factors"))

Excluded articles

**Table 10:** Excluded articles with reasons of exclusion

Reason for exclusion	Author (year)	Title	#
Less than 10 patients	Ron (1984)	Prevention of acute renal failure in traumatic rhabdomyolysis	1
Less than 10 patients	Uberoi (1991)	Acute renal failure in severe exertional rhabdomyolysis	2
Rhabdomyolysis was not confirmed	Shieh (1992)	Role of creatine phosphokinase in predicting acute renal failure in hypocalcemic exertional heat stroke	3
Less than 10 patients	Naqvi (1995)	Acute renal failure due to traumatic rhabdomyolysis	4
Hyperkalemia was reported among deceased patients.	Oda (1997)	Analysis of 372 patients with Crush syndrome caused by the Hanshin-Awaji earthquake	5
Only 3 patients were diagnosed with rhabdomyolysis	Hojs (1999)	Rhabdomyolysis and acute renal failure in intensive care unit	6
Less than 10 patients	Chang (2001)	Evaluation of the severity of traumatic rhabdomyolysis using technetium-99m pyrophosphate scintigraphy	7
Mixed caused of rhabdomyolysis	Hatamizadeh (2006)	Epidemiologic aspects of the Bam earthquake in Iran: the nephrologic perspective	8
Less than 10 patients	Altintepe (2007)	Early and intensive fluid replacement prevents acute renal failure in the crush cases associated with spontaneous collapse of an apartment in Konya	9
Less than 10 patients	Chunguang (2010)	Characteristics of crush syndrome caused by prolonged limb compression longer than 24 h in the Sichuan earthquake	10
Less than 10 patients	Bache (2011)	Late-onset rhabdomyolysis in burn patients in the intensive care unit	11
Less than 10 patients	Bartal (2011)	Crush syndrome: saving more lives in disasters: lessons learned from the early-response phase in Haiti	12
Less than 10 patients	Boulter (2011)	Acute renal failure in four Comrades Marathon runners ingesting the same electrolyte supplement: coincidence or causation?	13
Repeated database and results	Sever (2011)	Application of the RIFLE criteria in patients with crush-related acute kidney injury after mass disasters	14
Less than 10 patients	De Gracia-Nieto (2016)	Acute Renal Failure Secondary to Rhabdomyolysis as a Complication of Major Urological Surgery: The Experience of a High-Volume Urological Center	15
The total number of patients diagnosed with electrolyte imbalance was reported	Lydecker (2017)	A comparison of drug-related and other cause compartment syndrome	16
Only evaluated hyperkalemia among 5 patients.	Jabur (2018)	An Observational Epidemiological Study of Exercise-induced Rhabdomyolysis Causing Acute Kidney Injury: A Single-center Experience	17
The total number of patients diagnosed with electrolyte imbalance was reported	Navarrete (2018)	Hyperkalemia in electrical burns: A retrospective study in Colombia	18
Did not report the number of hyperkalemic patients	Arnautovic (2019)	Evaluation of clinical outcomes in hospitalized patients with exertional rhabdomyolysis	19

Results of risk-of-bias assessment

**Table 11:** Results of risk-of-bias assessment Question 1: appropriate sample frame, Question 2: sampling method, Question 4: study subjects’ description, Question 5: coverage of the identified sample, Question 6: valid method used in diagnosis of rhabdomyolysis, Question 6*: valid method used in diagnosis of electrolyte imbalance, Question 9: adequate response rate.

S. No.	Studies (Author, Year)	Q1	Q2	Q4	Q5	Q6	Q6*	Q9
1	An, 1984 [46]	Yes	No	No	Yes	No	Yes	No
2	Malik et al., 1993 [45]	No	Unclear	Yes	No	Yes	Yes	No
3	Knottenbelt, 1994 [44]	Yes	Unclear	No	No	Yes	Yes	Yes
4	Sinert et al., 1994 [43]	No	Yes	Yes	No	Yes	Yes	Yes
5	Naqvi et al., 1996 [42]	Yes	Unclear	No	Yes	Yes	Yes	No
6	Iskit et al., 2001 [41]	No	No	No	No	No	Yes	No
7	Erek et al., 2002 [40]	Yes	Unclear	No	Yes	Yes	Yes	No
8	Pocan et al., 2002 [39]	Yes	Unclear	No	Yes	Yes	Yes	No
9	Demirkiran et al., 2003 [38]	Yes	No	Yes	Yes	Yes	Yes	Yes
10	Sever et al., 2003 [37]	Yes	No	No	Yes	Yes	Yes	No
11	Gunal et al., 2004 [36]	Yes	Unclear	Yes	Yes	Yes	Yes	Yes
12	Aoki et al., 2007 [35]	Yes	No	No	Yes	No	Yes	Yes
13	Kang et al., 2008 [34]	Unclear	Unclear	No	Unclear	No	Yes	Yes
14	Li et al., 2009 [33]	Yes	Unclear	No	Yes	Yes	Yes	Yes
15	Ozturk et al., 2009 [32]	Yes	No	No	Yes	Yes	Yes	No
16	Bonomini et al., 2011 [31]	Yes	Yes	Yes	Yes	Yes	Yes	No
17	He et al., 2011 [30]	No	Yes	No	No	Yes	Yes	Yes
18	Rosedale et al., 2011 [29]	Yes	No	Yes	Yes	Yes	Yes	Yes
19	Zhang et al., 2013 [28]	No	Unclear	No	No	Yes	Yes	Yes
20	Guner et al., 2014 [27]	Yes	No	No	Yes	No	Yes	Yes
21	Hernández et al., 2015 [26]	Unclear	Unclear	No	Unclear	No	Yes	Yes
22	Omar et al., 2016 [25]	Yes	Unclear	No	Yes	No	Yes	No
23	Safari et al., 2017 [24]	Yes	No	Yes	Yes	Yes	Yes	Yes
24	Safari et al., 2017 [16]	Yes	No	Yes	Yes	Yes	Yes	Yes
25	Stewart et al., 2017 [23]	Yes	No	No	Yes	No	Yes	Yes
26	Thompson et al., 2018 [22]	Yes	Yes	Yes	Yes	Yes	Yes	Yes
27	Luetmer et al., 2020 [21]	Yes	Unclear	No	Yes	No	Yes	Yes
28	Thongprayoon et al., 2020 [20]	Yes	Unclear	No	Yes	No	Yes	Yes
29	Mao et al., 2021 [19]	Yes	Unclear	No	Yes	No	Yes	Yes
30	Webber et al., 2021 [18]	Yes	Yes	Yes	Yes	No	Yes	Yes
31	Alafifi et al., 2023 [17]	No	No	No	No	No	Yes	Yes
32	Lim et al., 2023 [15]	Yes	No	No	Yes	No	Yes	No

JBI critical appraisal checklist for studies reporting prevalence data

Note: Questions 3, 7, and 8 were not utilized

Question 1: Was the sample frame appropriate to address the target population? Patients diagnosed with traumatic rhabdomyolysis (of any etiology), crush syndrome, and crush injury

Question 2: Were study participants sampled in an appropriate way? The method used for sampling should be census or consecutive

Question 3: Was the sample size adequate? Given the emergency nature of the disease, we didn’t incorporate this question in our risk of bias assessment, nevertheless, we excluded articles that included less than 10 patients

Question 4: Were the study subjects and the setting described in detail? Age, sex, cause of rhabdomyolysis, and the country should be stated

Question 5: Was the data analysis conducted with sufficient coverage of the identified sample?

Question 6: Were valid methods used for the identification of the condition?

Regarding rhabdomyolysis diagnosis: Mild rhabdomyolysis: CPK 300-1000 IU/L, moderate rhabdomyolysis (crush injury): CPK 1000-5000 IU/L, severe rhabdomyolysis was defined as having a blood CPK level above 5000 IU/L - 10,000 IU/L, crush syndrome was defined as having blood CPK level > 5000-10,000 IU/L accompanied with systemic complication (AKI, sepsis, organ failure or respiratory failure).

Regarding electrolyte imbalance: Serum potassium normal range 3.5-5.5 mEq/dL, Serum sodium normal range 135-145 mEq/dL, Serum calcium normal range 8.6-10.3 mEq/dL, Serum phosphate normal range 2.5-4.5 mEq/dL

Question 7: Was the condition measured in a standard, reliable way for all participants? Overlap with question 9 (was not utilized)

Question 8: Was there appropriate statistical analysis? Since we just extracted the number of patients diagnosed with electrolyte imbalance and the total number of patients, this question wasn’t applicable.

Question 9: Was the response rate adequate, and if not, was the low response rate managed appropriately?

Sub-group meta-analyses
